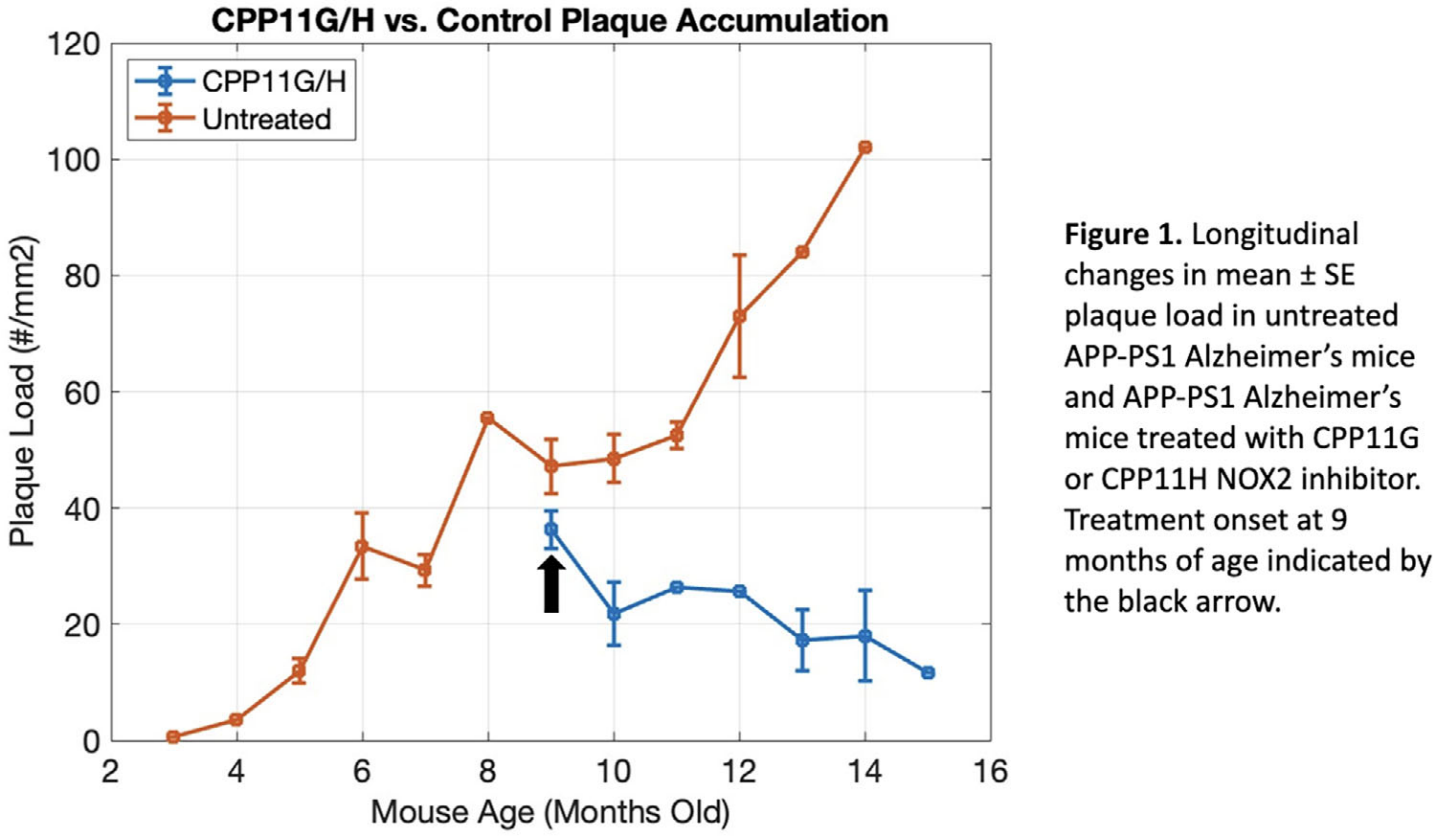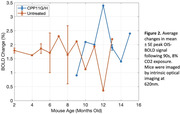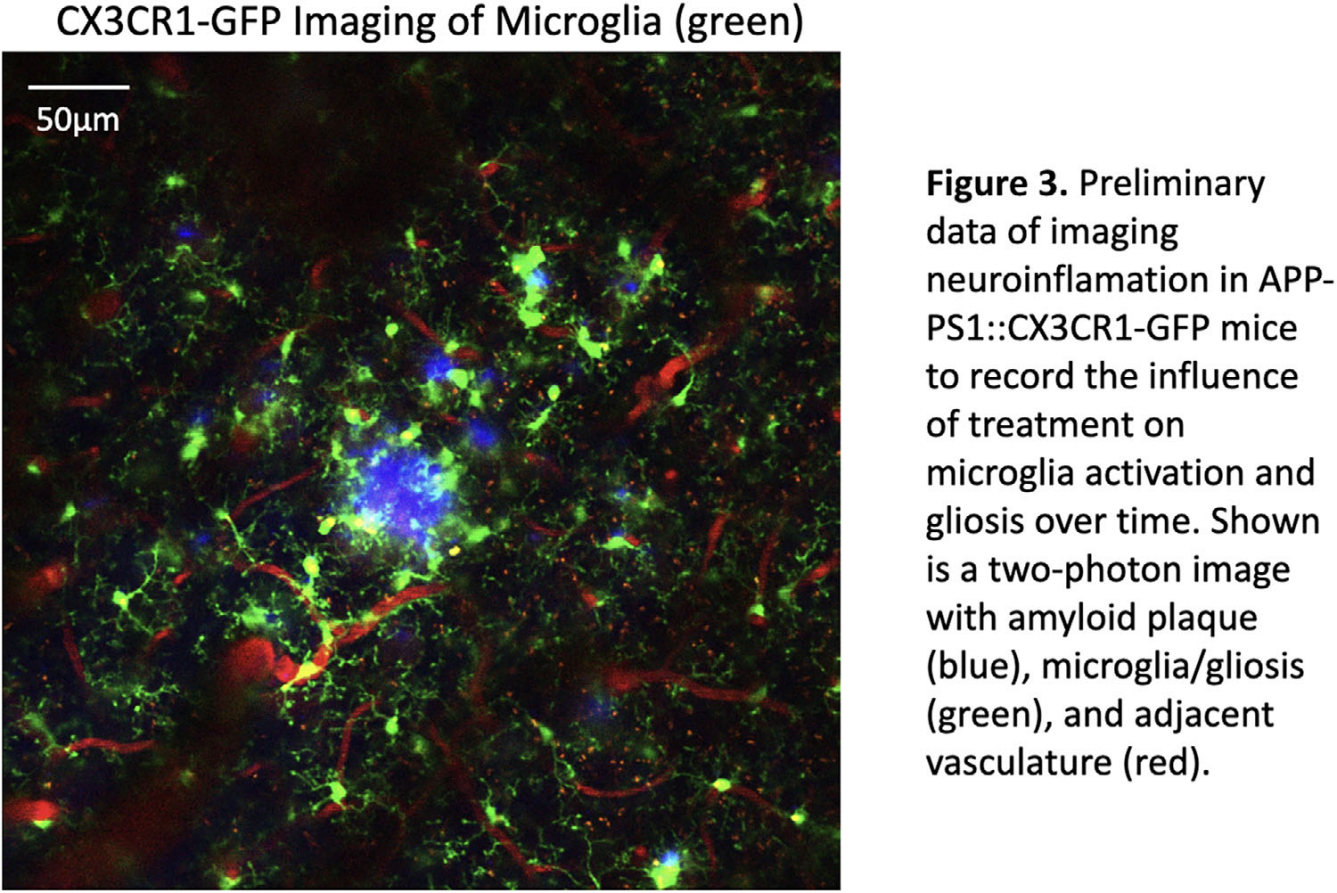# NADPH oxidase 2 small molecule inhibitors as a therapeutic strategy in the treatment of Alzheimer’s disease

**DOI:** 10.1002/alz.086492

**Published:** 2025-01-09

**Authors:** Jenna M Peretin, Christopher M Dustin, M. Eugenia Cifuentes‐Pagano, Patrick J. Pagano, Alberto L Vazquez

**Affiliations:** ^1^ University of Pittsburgh, Pittsburgh, PA USA

## Abstract

**Background:**

Alzheimer’s disease (AD) is a progressive neurodegenerative disease associated with neuroinflammation and heightened production of reactive oxygen species (ROS) in the brain from overactive NADPH Oxidase 2 (NOX2). The current study examines whether administration of a novel, brain‐penetrant NOX2 inhibitor (CPP11G & CPP11H) reduces amyloid plaque load and improves AD‐associated vascular dysfunction in a male APP‐PS1 mouse model of AD.

**Method:**

Intraperitoneal injections of CPP11G (n = 1) or CPP11H (n = 2) three times per week began at 9‐10 months of age in the treatment APP‐PS1 group (15 mg/kg). The control group of age‐matched APP‐PS1 mice received no treatment (n = 7). All mice were implanted with a 5mm cranial window for awake optical imaging at least 2 weeks prior to onset of treatment. Two‐photon microscopy was performed to assess longitudinal changes in amyloid plaque deposition after weekly Methoxy‐04 administration (1 mg/kg). Changes in plaque load were quantified weekly and averaged monthly. Additionally, wide‐field optical imaging at 620nm was performed with a 90s, 8% CO2 hypercapnic challenge. This imaging wavelength is sensitive to changes in blood oxygenation (OIS‐BOLD) to capture changes in vascular reactivity over time.

**Result:**

Treatment with CPP11G/H shows a trend toward reduction in cortical plaque load when compared to an untreated cohort (Fig. 1). OIS‐BOLD results for vascular reactivity studies show a slight trend of improved vascular function over time with CPP11G/H treatment (Fig. 2).

**Conclusion:**

Our preliminary findings show a strong trend in amyloid plaque reduction with ongoing CPP11G/H treatment in older‐aged AD mice and improved vascular function. Ongoing studies to increase the size of the treated cohort will reveal the reliability and statistical significance of CPP11G/H’s therapeutic benefits. We are also examining the impact of treatment on microglia activation and gliosis in APP‐PS1::CX3CR1‐GFP mice (Fig. 3).